# Spinal segmental myoclonus as an unusual presentation of multiple sclerosis

**DOI:** 10.1186/s12883-015-0271-y

**Published:** 2015-02-27

**Authors:** Raed Abdullah Alroughani, Samar Farouk Ahmed, Riyadh Ahmed Khan, Jasem Yousef Al-Hashel

**Affiliations:** Department of Medicine, Division of Neurology, Amiri Hospital, Arabian Gulf Street, Sharq, 13041 Kuwait; Department of Medicine, Neurology Clinic, Dasman Diabetes Institute, Kuwait, Kuwait; Department of Neurology, Ibn Sina Hospital, Kuwait, Kuwait; Department of Neurology and Psychiatry, Faculty of medicine, Al-Minia University, Minya, Egypt; Department of Medicine, Kuwait University, Kuwait, Kuwait

**Keywords:** Multiple Sclerosis, Myoclonus, Spinal cord, EMG

## Abstract

**Background:**

Unusual presentations of multiple sclerosis (MS) at onset may post a diagnostic dilemma to the treating neurologists. Spinal myoclonus is rare in MS and may lead to perform extensive investigations to rule out other etiologies affecting the spinal cord.

**Case presentation:**

We described a 31-year-old male who presented with involuntary brief jerky movements of the left shoulder and arm with significant wasting of shoulder muscles. In retrospect, the patient had a progressive right leg weakness one year prior to his presentation. Needle electromyography confirmed the presence of rhythmic irregular burst discharges in motor units of muscles expanding from the third to the sixth cervical region with normal nerve conduction parameters. There was no evidence of cortically generated myoclonic jerks using time-locked electroencephalogram. Magnetic Resonance Imaging of the brain and cervical cord along with the presence of oligoclonal bands in cerebral spinal fluid confirmed the diagnosis of MS. Based on the history and progressive clinical features, a diagnosis of primary progressive MS was established.

**Conclusion:**

Spinal myoclonus can be the presenting manifestation of MS in association with demyelinating plaques in the root exit zones of the spinal cord. Spinal myoclonus may pose a diagnostic challenge when it presented at the disease onset and especially in patients with progressive course at onset. Our patient represents the first reported primary progressive MS case in the literature with spinal myoclonus presentation.

## Background

Segmental myoclonus refers to involuntary brief rhythmic contraction of group of muscles supplied by one or more contiguous segments either in the brainstem or the spine (spinal segmental myoclonus) [1]. Spinal myoclonus can be caused by trauma, spondylosis, tumors, infections, myelitis, or ischemia [1,2]. We describe a patient with spinal segmental myoclonus as a rare presentation of multiple sclerosis (MS).

## Case presentation

A 31-year-old male soldier presented with a 2-month history of brief involuntary jerking of the left shoulder and arm, which persisted during sleep. In retrospective, he developed subacute weakness of his right lower limb one year ago. He had been using a cane to support his walking. He denied any associated neck pain, limb or facial parasthesia, bulbar or sphincteric symptoms. His past medical and family histories were unremarkable. At presentation, neurological examination revealed myoclonic jerks at left shoulder involving both agonist and antagonist muscles along with wasting of supraspinatus, infraspinatus, subscupularis, triceps, biceps, deltoid, and brachioradialis muscles. Weakness of proximal muscles of left upper limp (grade 4/5 on the Medical Research Council “MRC”) and distal muscles of right lower limb (grade 3/5 on MRC) were noted. There were bilateral leg spasticity and exaggerated deep tendon reflexes and extensor planters. Cerebellar and sensory examination were unremarkable apart from positive Romberg’s. Gait was spastic.

**Figure 1:**
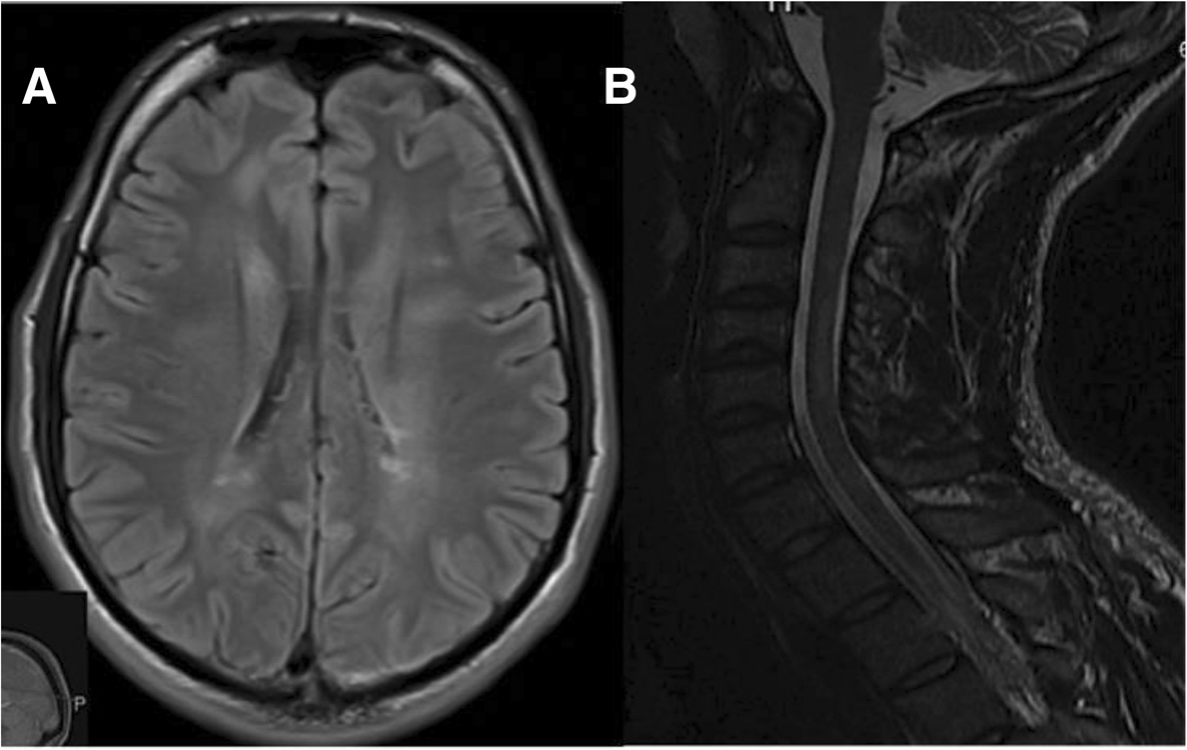
**MRI brain and cervical spine images. A)** Axial Fluid-Attenuated Inversion Recovery (FLAIR) image of the brain showing demyelinating plaques in periventricular and juxtacortical regions; **B)** Sagittal T2 image of the cervical spine showing intramedullary demyelinating plaques at C3 and C4-C5 levels.

Routine hematological and biochemical laboratory investigations including serum calcium, copper, ceruloplasmin levels, thyroid hormone levels, and sedimentation rates were within normal limits. MRI brain and spine revealed multiple demyelinating lesions in the brain, cervical and thoracic spine satisfying Barkoff criteria [Figure 1]. There were intramedullary demyelinating plaques at C3 and C4-5 spinal levels corresponding to the involuntary movements. Cerebrospinal fluid (CSF) revealed normal cell counts, protein and glucose but positive for oligoclonal bands. Secondary causes of myoclonus such as infectious disease (HIV, HSV, syphilis, HTLV 1 & 2) were excluded. Autoimmune profile including ANA, ENA, and serum ACE along with screening tests for leukodystrophies and paraneoplastic disorders were negative. Nerve conduction study (NCS) was within normal limits whereas needle electromyography (EMG) revealed rhythmic irregular burst discharges with a rate of 1–3 Hz in motor units of muscles expanding from the third to the sixth cervical region. Electroencephalogram was normal. Visual-evoked potential (VEP) was delayed in both eyes and somatosensory evoked potentials (SSEP) showed low cervical but well-defined cortical responses after stimulation from the left side.

The history along with the involvement of multiple neuroaxis on clinical examination and paraclinical tests, were supportive of a demyelinating disorder. The patient was diagnosed as primary progressive multiple sclerosis (PPMS) according to the revised 2010 McDonald diagnostic criteria [3]. The patient refused treatment with intravenous methylprednisolone and elected to have symptomatic treatment. He showed a good response to oral levetiracetam 500 mg twice daily and oral baclofen 10 mg twice daily in helping myoclonus and spasticity respectively.

## Discussion

We described an unusual presentation of spinal myoclonus in our patient as part of a progressive demyelinating disease (PPMS). The initial presentation was due to partial myelitis resulting in leg weakness one year prior to the onset of myoclonic jerks. MS demyelinating plaques at root exit zones involving contiguous spinal segments can cause spinal segmental myoclonus. In our case, there was no evidence of this being cortical in origin given the absence of time-locked cortical correlates in the back-averaged EEG activity preceding spontaneous jerks, and cortical response in SSEPs were of normal amplitude. Propriospinal myoclonus is an additional form of spinal myoclonus that had been described in the literature in which extensive contraction of axial and trunk muscles through as slowly conducting propriospinal pathways [4,5]. Both spinal segmental and propriospinal terms may be intermixdly used when continuous groups of axial and proximal limb muscles were affected.

The pathophysiology of spinal myoclonus is poorly understood. Proposed mechanisms included the loss of inhibitory function of local dorsal horn interneurons, abnormal hyperactivity of local anterior horn neurons, aberrant local axons re-excitations and loss of inhibition from supra-segmental descending pathways [1,5,6]. Demyelinated axons can be abnormally hyper-excitable and can display spontaneous discharges, which alone, or driven reflexibly, could lead to myoclonus [7]. In our case, the presence of cervical lesions at the root exit zones may result in disinhibition of alpha motor neurons and disruption of spinal interneurons circuits leading to the development of myoclonus.

Only a handful of MS cases were reported in the literature along with other demyelinating disorders as shown in Table 1. In 1986, Jancovic and Pardo reported a series of 19 patients with segmental myoclonus. Six patients had demyelinating disorder; of whom one had a spinal myoclonus while the rest had brainstem myoclonus [1]. Another case presented with right arm and upper trunk myoclonus was described in 1992 and found to be due to demyelinating plaques at C3-C4. The authors concluded that the case represented a propriospinal pattern [4]. Khafizova et al. described a similar case with spinal segmental myoclonus caused by a cervical cord lesion which turned by be MS [8]. Spinal myoclonus was reported in other demyelinating disorders such as ADEM [9], NMO [10,11] and idiopathic transverse myelitis [12]. Nevertheless, our patient is the first PPMS case to be reported in the literature with a presentation of spinal segmental myoclonus.

**Table 1:** **List of the reported cases of spinal myoclonus due to demyelinating disorders in the literature**

**No**	**Age/gender**	**Diagnosis**	**Predominant site of myoclonus**	**Muscle involved**	**Location of MRI lesion**	**Author, year**
1	13/F	MS	Left arm	Triceps bicep, brachioradialis	-	Jankovic et al., 1986 [1]
2	23/F	MS	Right arm/shoulder	Latissimus dorsi, deltoid, triceps, SCM, trapezius	C2-C4	Kapoor et al., 1992 [4]
3	-	MS	-	-	Cervical cord	Khafizova et al., 2014 [8]
4	7/M	ADEM	Left arm	Upper paraspinal	C1-2	Kabakus et al., 2006 [9]
5	59/F	NMO	Right leg	-	-	De Mattos et al., 1993 [10]
6	32/F	NMO	Axial	Rectus abdominis, SCM	T8-T10	Vetrugno et al., 2009 [11]
7	12/M	Transverse Myelitis	Right leg	Quadriceps, hamstring	Normal	Keswani et al., 2002 [12]

ADEM: Acute disseminated encephalomyelitis; SCM: Sternocleimastoid; MS: Multiple Sclerosis; NMO: Neuromyelitis Optica.

## Conclusion

Segmental spinal myoclonus caused by MS demyelinating plaques could represent a diagnostic challenge at the time of presentation and might lead to exhaustive investigations to exclude other causes of myoclonus.

### Consent

Written informed consent was obtained from the patient for publication of this Case report and any accompanying images.

## Abbreviations

ADEM: Acute disseminated encephalomyelitis
CSF: Cerebral spinal fluid
EEG: Electroencephalogram
EMG: Electromyography
MRC: Medical research council
MRI: Magnetic resonance imaging
MS: Multiple sclerosis
NCS: Nerve conduction study
NMO: Neuromyelitis optica
SSEP: Somato-sensory evoked potential
VEP: Visual evoked potential
